# Relationship Between Family Functioning and Mental Health Considering the Mediating Role of Resiliency in Type 2 Diabetes Mellitus Patients

**DOI:** 10.5539/gjhs.v7n3p254

**Published:** 2014-11-30

**Authors:** Mostafa Bahremand, Alireza Rai, Mostafa Alikhani, Samira Mohammadi, Karoon Shahebrahimi, Parisa Janjani

**Affiliations:** 1 School of Medicine, Kermansh University of Medical Sciences, Kermansh, Iran; 2 School of Pharmacy, Kermansh University of Medical Sciences, Kermansh, Iran

**Keywords:** diabetes mellitus, family functioning, mental health, patients, resiliency

## Abstract

This study was aimed at describing the mediating role of resiliency in the relationship between family functioning and mental health in patients with type 2 diabetes mellitus. This descriptive research was a correlational study. A total of 225 individuals were chosen by simple random sampling technique from type 2 diabetic patients presented to diabetes care centers in Kermanshah in 2014 in Iran. The 12-item General Health Questionnaire (GHQ-12), the Family Assessment Device (FAD) and the Resilience Scale (CD-RISC) were used to collect the required data. The collected data were analyzed using the Pearson’s correlation test and to study the mediating role of resiliency in family functioning and mental health interaction, the path analysis method was applied. The results showed that there is a relationship between family functioning, resilience and mental health. Resilience plays a mediating role between family functioning and mental health. Therefore, paying attention to resilience in patients may lead to improving mental health in diabetic patients.

## 1. Introduction

The worldwide epidemiology of type 2 diabetes has raised this disease as a major public health problem. Considering the worldwide prevalence rate of diabetes, in 2010 it was estimated that 6.4% of adults suffer from type 2 diabetes mellitus. The prevalence rate of T2D is projected to rise by 7.7% of the total world population or to over 439 million adults until 2030 (Shaw et al., 2010). According to the statistics released by the Iranian Diabetes Society in 2006, over 4 million people were diagnosed with T2D and the annual growth rate was expected to increase by 1% (Esmailpoor et al., 2011). T2D cause many limitations for the sufferer such as requiring repeated insulin injections, bearing the costs of insulin injections, need to follow a diet plan, recurrent infections and possibility of repeated hospitalizations (Livneh & Wilson, 2003). These cause depression, and have negative impact on interpersonal and family relationships, social associations, and on the whole, the general health and psychological well-being of the patients (Skandarian et al., 2009). The previous studies show that the prevalence rate of mental illness in diabetic patients is higher than in non-diabetic subjects (Jimenez-Garcia et al., 2012; Serious psychological, 2004), and this prevalence rate was reported as up to 41.5% in patients newly diagnosed with T2D (Rane et al., 2011). The research conducted by Brown and Nichols (2004) showed that the mental health status of diabetic patients is lower as compared to that of non-diabetic individuals (Nichols & Brown, 2004). The comorbidities of diabetes and psychiatric disorders has a negative effect on aggravation of symptoms, development of various adverse complications, reduced response to treatment, and even higher mortality rate in diabetic patients (Atadokht et al., 2013). Given that general health status plays a major role in the lives of patients with diabetes, further attempts have been made to recognize structures affecting and promoting the mental health of these patients. From amongst contributing factors to mental health status, it is possible to point out family and resilience.

Family functioning is one of the important aspects of family environment which may affect the physical, social and emotional health of individuals. In fact, what happens within a family and how the family functions are the crucial factors in creating flexibility and mitigating current and future risks associated with unfortunate events and unsuitable conditions (Gamari & Khoshnam, 2011). Research evidence shows that family functioning has a strong association with metabolic control (glycosylated hemoglobin) and health of children with DM (Cohen et al., 2004, Maharaj et al., 2004; Lewin et al., 2006). Furthermore, it has been proved that there is a significant relationship between poor family functioning and suffering from physical symptoms, anxiety, sleep disorder, depression and disruption to normal social functioning (Zargar et al., 2007).

Many researchers have reported a significant and negative relationship between resilience and psychological problems, expressing that this structure can take on a mediating role between mental health and many other variables, and that individuals will be able to increase their stress and anxiety tolerance and resist and overcome many causative factors of psychological problems by developing their resilience (Agaibi & Wilson, 2005; Joseph et al., 1997; Connor, 2006; Inzlicht et al., 2006; Pinquart, 2009; Besharat, 2007). Resiliency helps people use their capabilities to achieve success and growth in their personal lives in spite of the risk factors involved and in difficult circumstances and take as an opportunity for rising to these challenges aiming at strengthening themselves (Garbowski, 2010).

Given the increasing prevalence of diabetes worldwide, living with T2D should also be considered. So far, studies suggest that these patients and also their families need extensive non-pharmacological interventions due not only to the higher prevalence of psychological disorders, but also to create better cooperation and compliance in the treatment process at various stages so as to avoid long-term complications of the disease and also enjoy a higher quality of life while coping with the disease. As noted above, family functioning can influence the mental health status of individuals, but the question then arises as to how family functioning exerts an influence on mental health status? It seems that resiliency can assume a mediating role between family functioning and mental health status. Thus, the purpose of this study was to explain the mediating role of resilience in the relationship between family functioning and mental health status of patients with type 2 DM.

## 2. Materials and Methods

This study was a descriptive research of correlational type. The population in this research consisted of all type 2 diabetic patients (aged 35 to 68 years), who presented to diabetes care centers of Kermanshah in 2014 with at least a 5-year history of T2D and no previous record of mental illness. A sample size of 225 individuals was chosen by employing the simple random sampling technique. The selected patients were asked to fill in the questionnaires after informed consent was obtained for participating in the study and the confidentiality of their information was guaranteed. The questionnaires were collected after being individually completed by the patients in the presence of the researcher. Considering the possibility of having some drop outs in the research and/or inappropriate completion of some questionnaires, a sample group of 250 individuals was selected, of which a sample size of 225 individuals was included. Statistical analyses were done by the SPSS Software for Windows (ver. 19.0) and AMOS (ver. 18.0) using the Pearson correlation coefficient test and path analysis. Descriptive indices including mean and its standard deviation were used to express data.

### 2.1 Research Tool

#### 2.1.1 12-item General Health Questionnaire (GHQ-12)

This questionnaire was introduced by Goldberg in 1972 for identifying psychiatric disorders within community or various clinical settings. It asks whether the patients has recently experienced a particular symptom (like abnormal feelings or thoughts) or changes in the observable aspects of their behavior in the period of recent four weeks. Therefore, the questions emphasize on the here-and-now aspect of the response. Many researchers (Worthington, 2000) have admitted that the GHQ is one of the most widely recognized screening tools for psychiatry, behavioral science and psychology, which has had a profound impact on the development of research activities. The GHQ has different versions, namely GHQ-12, GHQ-28, GHQ-30 and GHQ-60, among which the 12-item and 28-item versions have been the most extensively used screening instruments in Iran and other countries. The GHQ-12 is composed of 12 items out of the original GHQ containing 60 items and is used to identify the severity of psychological distress experienced by an individual within the past few weeks (Handerson & Verhak, 1990). This questionnaire consists of four subscales including physical symptoms, anxiety, social dysfunction, and depression. There are two common scoring methods for the GHQ. The first technique is bi-modal (0-0-1-1) based on Goldberg’s original scoring method, in which response categories of A and B receive score of zero and categories of C and D receive score of 1.0. This gives scores ranging from 0 to 12. The other scoring method is Likert scoring style, in which response categories (A, B, C, and D) receive scores of 0, 1, 2, and 3, respectively. The GHQ-12 gives a total score of 36 with the latter method of scoring. Goldberg (1972) assessed the validity of the 60-item GHQ in his initial study and reported sensitivity, specificity, and overall misclassification as 77.5%, 88.4%, and 15.4%, respectively by using Likert scoring method. These values by using Likert scoring method were respectively 80.6%, 93.3%, and 10.9%. In a recent study on junior students in a university, these values were reported respectively as 62%, 65%, and 35.7% for GHQ-12 (Goldberg & Williams, 1988). In this study, reliability of the questionnaire was also evaluated and its Cronbach’s alpha was calculated as 0.80.

#### 2.1.2 The Connor-Davidson Resilience Scale (CD-RISC)

This scale was developed by Connor and Davidson (2003) as a measure of stress coping ability. The developers of this scale believed that this questionnaire can properly separate resilient individuals from the non-resilient in clinical and non-clinical groups and is capable of being administered to research and clinical situations (Rahimian Boger & Nejadfarid, 2008). This scale comprises of 25 items, each rated on a 5-point Likert scale (0-4): “Never”, “Rarely”, “Sometimes”, “Often” and “Always”. Its possible minimum score is zero and its maximum score is 100. The reliability and validity of the Persian version of the CD-RISC were evaluated and established in the primary studies on normal subjects and patient (Besharat 2007). This scale was standardized by Mohammadi (2005) in Iran (Mohammadi 2005). In a study conducted by Samani, Jokar and Sahragard (2007), Cronbach’s alpha reliability coefficient was calculated as 0.87 (Samani et al. 2006). In this study, Cronbach’s alpha of the questionnaire was assessed as 0.89.

### 2.2 Family Assessment Device (FAD)

The FAD is a 53-item questionnaire and was developed based on the McMaster model of family functioning. This device was developed by Epstein, Baldwin & Bishop (1983) aiming at collecting information on various structural and organizational dimensions of the family system. This questionnaire is a self-report measure assessing and evaluating family functioning and quality of interaction among its members, and its items are separately rated on a 5-point Likert scale: “Very rarely”, “Rarely”, “Occasionally”, “Frequently” and “Very frequently”. The FAD is made up of seven subscales, namely “Communication”, “Affective Involvement”, “Role Playing”, “General Functioning”, “Problem Solving”, “Affective Responsiveness” and “Behavior Control”. FAD total score may be computed from adding together all the seven subscale scores. The validity and reliability of the FAD were evaluated (after being developed by Epstein et al. in 1983) using a sample of 503 individuals. The Cronbach’s alpha reliability coefficient of the sets falls within the range of 0.72 to 0.92 indicating a high degree of internal consistency. Studies carried out by Bokharian (2002), Norouzi (1998), Mollataghi (1998), Bahari (2000) and Amini (2000) in Iran indicate that the reliability and validity of this questionnaire is high. Bokharian (2002) reported Cronbach’s alpha reliability coefficients of certain subscales including problem-solving, emotional expression, and general functioning as 0.92, 0.75 and 0.93, respectively (Saatchi et al., 2010). The Cronbach’s alpha reliability coefficient of this questionnaire was was 0.91 in this study.

## 3. Results

Table 1 presents mean, standard deviation (SD), and correlation coefficient of the studied variables.

**Table 1:** Mean, standard deviation, and correlation coefficients between variables

	Mean (SD)	1	2	3
**Mental health**	15.825.94)	1	- 0.60**	- 0.53**
**Resiliency**	61.89 (14.68)	- 0.60**	1	0.53**
**Family functioning**	132.59 (26.06)	- 0.53**	0.53**	1

*P< 0.05;

** P< 0.01.

To study the mediating role of resiliency in family functioning and mental health interaction, the path analysis method was applied. The findings demonstrated that the direct relationship between family functioning and resiliency was 0.57 and between resiliency and mental health this value was –0.48. Also, the direct relationship between family functioning and mental health was –0.27. All these coefficients were significant at P<0.01. Direct standardized and unstandardized coefficients between variables are shown in Table 2.

**Table 2:** Direct standardized and unstandardized coefficients between the variables

	Standardized coefficients	Unstandardized coefficients	Significance level
**Family functioning-resiliency**	0.57	0.24	0.001
**Resiliency-mental health**	- 0.48	- 0.25	0.001
**Family functioning-mental health**	- 0.27	- 0.06	0.001

The results showed that standard coefficient of the relationship between family functioning and mental health via resiliency was –0.27 which was significant at P<0.01.

**Table 3:** Indirect standardized and unstandardized coefficients between the variables

	Standardized coefficient	Unstandardized coefficient	Significance level
**Family functioning with mental health through resiliency**	- 0.27	- 0.06	0.001

**Figure 1:**
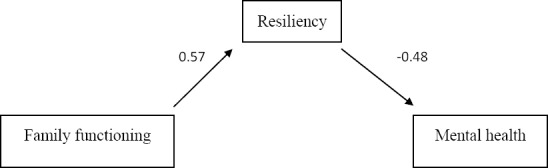


## 4. Discussion

This study was aimed at describing the mediating role of resiliency in the relationship between family functioning and mental health in patients with T2D. The results obtained showed that there is a negative relationship between resilience and mental health. Considering the type of questionnaire used in this study, this result implies that high levels of resilience result in improvement of mental health and reducing the high risk of psychological disorder. Conversely, low levels of resilience is associated with decline in the mental health of diabetic patients. In this part, the results of this study are consistent with those of the previous studies (Agaibi & Wilson, 2005; Joseph et al., 1997; Connor, 2006; Inzlicht et al., 2006; Pinquart, 2009; Besharat, 2007), Considering the fact that resilience is defined as the ability and potentiality of an individual for achieving good health, overcoming the disease and achieving personal and social growth and making use of these conditions and challenges as an opportunity to further empower They under difficult and particularly stressful circumstances, This result is completely explainable. That is to say, the high level of resilience in diabetic Patients protects them against damages to their mental health under disease conditions and despite the functional restraints imposed on their lives. In fact, resilience is best defined as an individual’s ability to properly adapt to stress and adversity, and diabetic patients need to cope with complications caused by T2D and its associated limitations. The results of this study showed that there is a negative relationship between family functioning and mental health in T2D patients; considering the type of research tools and techniques, this result implies that good family functioning results in better mental health of the individual and poor family functioning results in the decline of the individual’s mental health. In this part, the results of this study are consistent with those of the previous studies (Cohen et al., 2004; Maharaj et al., 2004; Lewin et al., 2006; Zargar, 2007). In view of the fact that T2D may impose limitations on the sufferers such as requiring repeated insulin afford the costs of insulin injections injections, need to follow a diet plan, recurrent infections and possibility of repeated hospitalizations (Livneh & Wilson, 2003), which affect the whole family, these conditions may cause a diabetic patient less harm and cause no damage to his/her mental health, if living in a family that functions properly. Considering that the main function of a family is to meet individual needs of the family members and also the fact that these needs may turn out to be further demands for family resources and time under such circumstances that one of the family members is suffering from a chronic disease, such as diabetes; in cases where the family is capable of responding to these needs, the consequences of diabetes may cause less harm to the individual, and he/she may enjoy better mental health.

This study showed that in addition to existence of a direct relationship between resilience, family functioning and mental health, there is also an indirect relationship between family functioning and mental health through resiliency. The relationship between familyfunctioning and mental health through resiliency is -0.27; in other words, resilience is affected by family functioning and mental health by resilience, and the relationship between family functioning and mental health is enhanced through resilience. In this part, the results of this study are consistent with those of the previous studies (Besharat, 2007; Garbowsk, 2010). The results of this study indicate that resilience is more important than family functioning so far as mental health is concerned, and that mental health is rather affected by resilience than the family functioning; people with high resilience have considerable potential to gain social and family support, develop a better relationship with their family members, avoid diabetes burnout and enjoy better mental health. In fact, The high performance of the family functioning can help develop the individual resilience, and an individual can enjoy better health, if showing greater resilience.According to the results of this study, it is possible to conclude that diabetic patients have greater resilience if enjoying better family functioning, and that the greater the individual’s resilience, the better mental health he/she will have. Therefore, it is important to allow for the major role of resilience when treating diabetic patients, and that it is essential not to neglect such a prominent role. Other studies (Agaibi & Wilson, 2005; Joseph et al., 1997; Connor, 2006; Inzlicht et al., 2006; Pinquart, 2009; Besharat, 2007), have also proved that promoting resilience in individuals can enhance resistance to deleterious effects of anxiety and stress factors as well as causes of various psychological disorders. This study was conducted on diabetic patients in Kermanshah; thus it is recommended to proceed with caution when generalizing the results. Therefore, it is suggested to conduct this study in other societies in order to compare the results
